# NLRP3 activation in endothelia promotes development of diabetes-associated atherosclerosis

**DOI:** 10.18632/aging.103666

**Published:** 2020-09-23

**Authors:** Dong Huang, Wei Gao, Xin Zhong, Junbo Ge

**Affiliations:** 1 Department of Cardiology, Zhongshan Hospital, Fudan University, Shanghai Institute of Cardiovascular Diseases, Shanghai 200032, China

**Keywords:** atherosclerosis, diabetes, endothelial cells, NLRP3

## Abstract

Inflammatory damage to endothelial cells plays a pivotal role in the diabetes-provoked atherosclerosis (AS). PYD domains-containing protein 3 (NLRP3) induces formation of inflammasome activates caspase-1, which subsequently cleaves the precursor form of IL-1β (pro-IL-1β) into the processed, secreted form IL-1β to promote the immune responses in AS. However, it is not known whether NLRP3 activation specifically in endothelial cells causes AS. Here, in an in vitro model for AS, we showed that NLRP3-depleted human aortic endothelial cells (HAECs) became resistant to apoptotic cell death, maintained proliferative potential and reduced reactive oxygen species (ROS) production upon treatment with oxidized low-density lipoprotein (ox-LDL). Next, the role of NLRP3 in endothelial cells in the development of diabetes-associated AS was assessed in endothelial cell-specific NLRP3 mutant, ApoE (-/-) mice (APOEKO/Tie2p-Cre/NLRP3^MKO^), compared to control ApoE (-/-) mice (APOEKO), supplied with either high-fat diet (HFD), or normal diet (ND). We found that endothelia-specific NLRP3-depletion significantly attenuated AS severity in mice treated with HFD, likely through reduced apoptotic death of endothelial cells and production of ROS. Together, our data suggest that NLRP3 activation in endothelial cells promotes development of diabetes-associated AS.

## INTRODUCTION

Diabetic status is known to promote the development of atherosclerosis (AS) [1], in which endothelial cells under inflammatory attacks are progressively damaged [2–4].

A proinflammatory cytokine, interleukin 1β (IL-1β), plays a profound role in a variety of immune diseases. The elucidated mechanisms by which the biologically active IL-1β is made in the cell usually involve activation of a protein complex called the inflammasome [5]. Inflammasome is typically formed by a NLR (Nod-like receptor) family member that oligomerizes the apoptosis-associated speck-like protein containing a caspase-recruitment domain (ASC) to dimerize and activate caspase-1, which subsequently cleaves the precursor form of IL-1β (pro-IL-1β) into the processed, secreted form IL-1β [6]. PYD domains-containing protein 3 (NLRP3) is the most important enzyme [7]. However, it is not known whether NLRP3 activation specifically in endothelial cells may cause AS. This question was addressed in the current study using endothelia-specific NLRP3 mice by breeding inducible NLRP3 mutant mice with mice carrying a Cre recombinase under an endothelia-specific Tie2 promoter (Tie2p-Cre).

Apolipoprotein E (ApoE) is a strong suppressor for AS [8–10]. ApoE-knockout [ApoE (-/-)] mice develop chronic inflammation in response to diet-induced hypercholesterolemia [8, 9] and AS features. Hence, a high fat diet (HFD) has been commonly used to reproducibly induce AS in ApoE (-/-) mice [11–13].

Here, in an in vitro model for AS, we showed that NLRP3-depleted human aortic endothelial cells (HAECs) became resistant to apoptotic cell death, maintained proliferative potential and reduced reactive oxygen species (ROS) production upon treatment with oxidized low-density lipoprotein (ox-LDL). Next, the role of NLRP3 in endothelial cells in the development of diabetes-associated AS was assessed in endothelia-specific NLRP3 mutant, ApoE (-/-) mice (APOEKO/Tie2p-Cre/NLRP3^MKO^), compared to control ApoE (-/-) mice (APOEKO), supplied with either HFD, or normal diet (ND). We found that endothelia-specific NLRP3-depletion significantly attenuated AS severity in mice treated with HFD, likely through reduced apoptotic death of endothelial cells and production of ROS.

## RESULTS

### Knockdown of NLRP3 in HAECs

**Figure 1:**
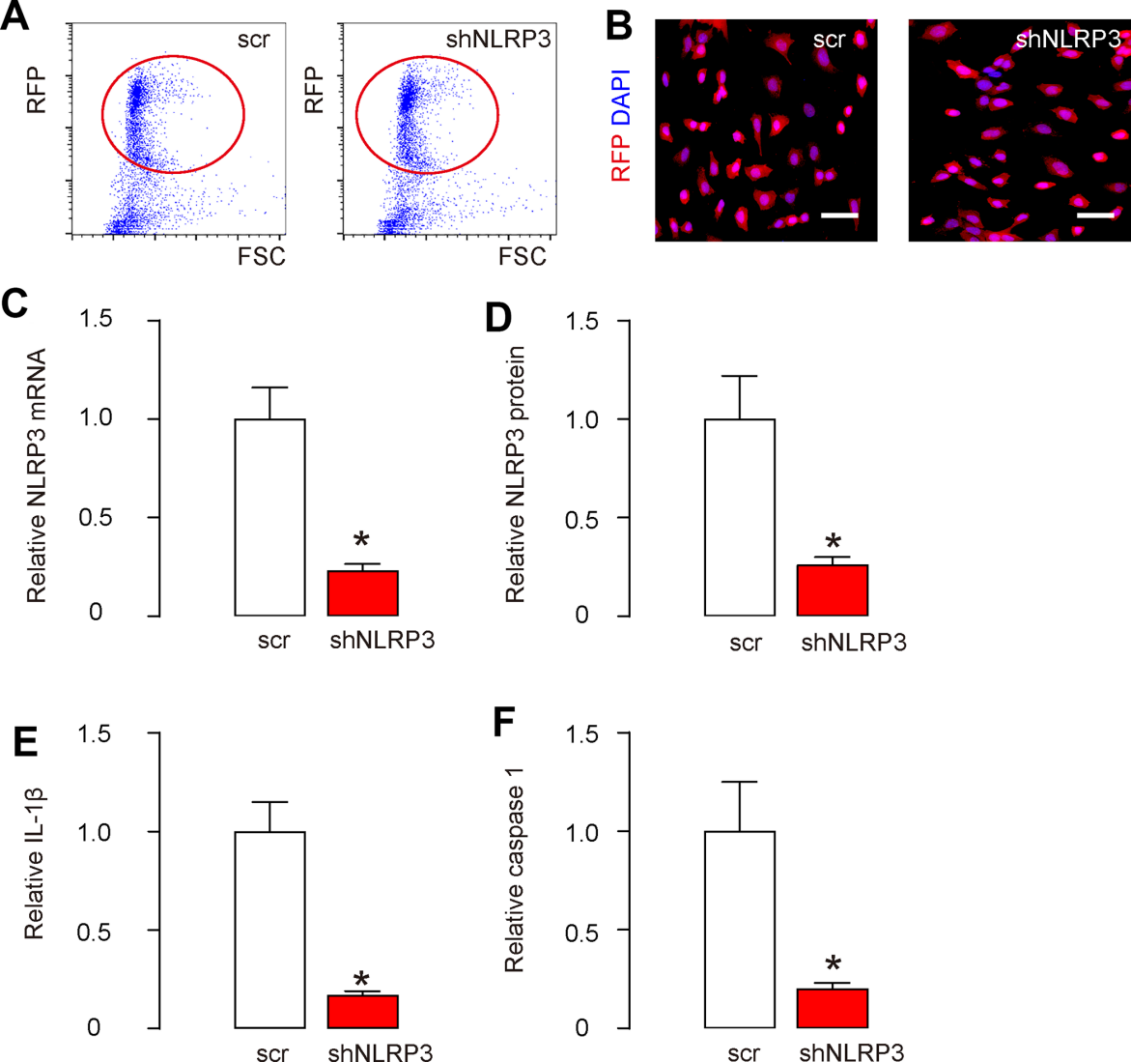
**Knockdown of NLRP3 in HAECs.** HAECs were transfected with either a scramble sequence (scr), or a short hairpin small interfering RNA for NLRP3 (shNLRP3). (**A**) All plasmids also carried a red fluorescent protein (RFP), which allowed the transfected cells to be visualized and isolated by flow cytometry based on RFP, shown by representative flow charts. (**B**) The purified transfected cells were visualized due to RFP in culture. (**C**, **D**) NLRP3 levels in purified cells by RT-qPCR (**C**) and by ELISA (**D**). (**E**, **F**) IL-1β (**E**) and caspase 1 (**F**) levels in purified cells by ELISA. *p<0.05. N=5. Scale bars are 20 μm.

In order to understand whether NLRP3 activation specifically in endothelial cells may cause AS, first we did an in vitro study. HAECs were transfected with plasmids carrying either a scramble sequence (scr), or a short hairpin small interfering RNA for NLRP3 (shNLRP3). All plasmids also carried a red fluorescent protein (RFP), which allowed the transfected cells to be visualized and isolated by flow cytometry based on RFP (Figure 1A). The purified transfected cells were red fluorescent in culture (Figure 1B). Next, we tested the degree of knockdown of NLRP3 in HAECs. RT-qPCR showed that NLRP3 mRNA levels decreased by about 75% in shNLRP3-transfected cells (Figure 1C). Moreover, ELISA showed NLRP3 protein levels decreased by about 72% in shNLRP3-transfected cells (Figure 1D). The downstream targets of NLRP3, IL-1β (Figure 1E) and caspase 1 (Figure 1F), were both reduced in shNLRP3-transfected cells. These data confirmed a regulatory axis of NLRP3/ caspase-1/IL-1β, and validated the knockdown efficiency of shNLRP3.

### Responses of HAECs to Ox-LDL treatment are NLRP3 dependent

**Figure 2:**
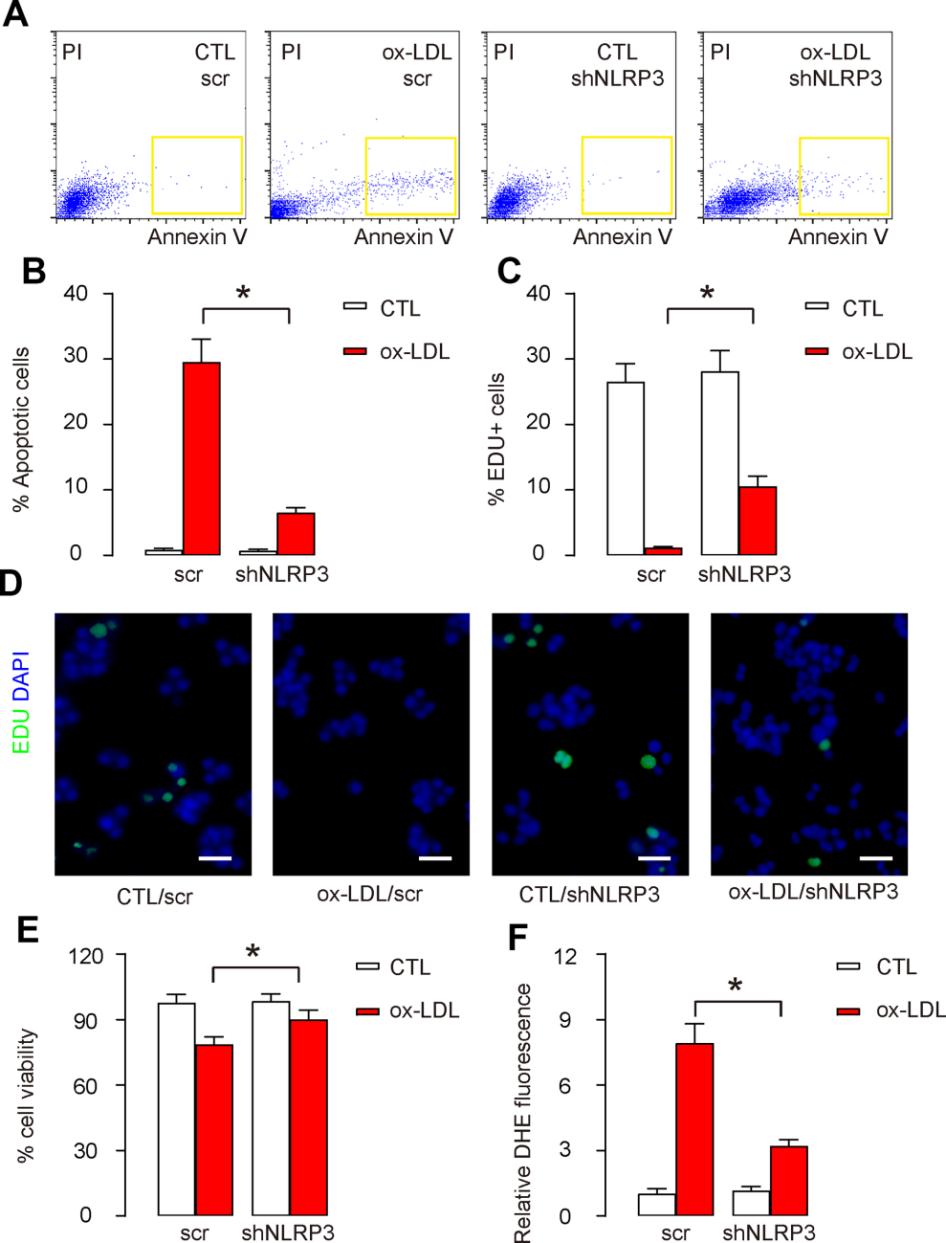
**Responses of HAECs to Ox-LDL treatment are NLRP3 dependent.** Next, shNLRP3-HAECs and scr-HAECs were challenged in an in vitro AS model by treatment with 100μg/ml oxidized low-density lipoprotein (ox-LDL). (**A**, **B**) Analyses of cell apoptosis by FITC Annexin V Apoptosis assay, shown by representative flow charts (**A**), and by quantification (**B**). (**C**, **D**) Analyses of cell proliferation by Edu assay, shown by quantification (**C**), and by representative images (**D**). (**E**) Quantification of viable cell number in an CCK-8 assay. (**F**) DHE assay for ROS. *p<0.05. N=5. Scale bars are 20 μm.

Next, shNLRP3-HAECs and scr-HAECs were challenged in an in vitro AS model using oxidized low-density lipoprotein (ox-LDL). In control scr-HAECs, ox-LDL induced significant increases in apoptotic cell death, shown by representative flow charts (Figure 2A), and by quantification (Figure 2B). On the other hand, basal apoptotic cell death did not alter in shNLRP3-HAECs, while ox-LDL-induced increases in apoptotic cell death were significantly attenuated in shNLRP3-HAECs, shown by representative flow charts (Figure 2A), and by quantification (Figure 2B). Cell proliferation was also assessed by Edu assay. In control scr-HAECs, ox-LDL induced significant decreases in cell proliferation, shown by quantification (Figure 2C), and by representative images (Figure 2D). On the other hand, basal cell proliferation did not alter in shNLRP3-HAECs, while ox-LDL-induced decreases in cell proliferation were significantly attenuated in shNLRP3-HAECs, shown by quantification (Figure 2C), and by representative images (Figure 2D). The effects of shNLRP3 on cell apoptosis and proliferation altered total cell number correspondingly in an CCK-8 assay (Figure 2E). ROS production was also assessed. In control scr-HAECs, ox-LDL induced significant increases in ROS production (Figure 2F). On the other hand, shNLRP3-HAECs did not alter basal ROS production, while ox-LDL-induced increases in ROS production were significantly attenuated in shNLRP3-HAECs (Figure 2F). Together, these data suggest that the effects of Ox-LDL treatment on HAECs are NLRP3 dependent.

### Preparation of endothelia-specific NLRP3 mutant mice in ApoE-null background

**Figure 3:**
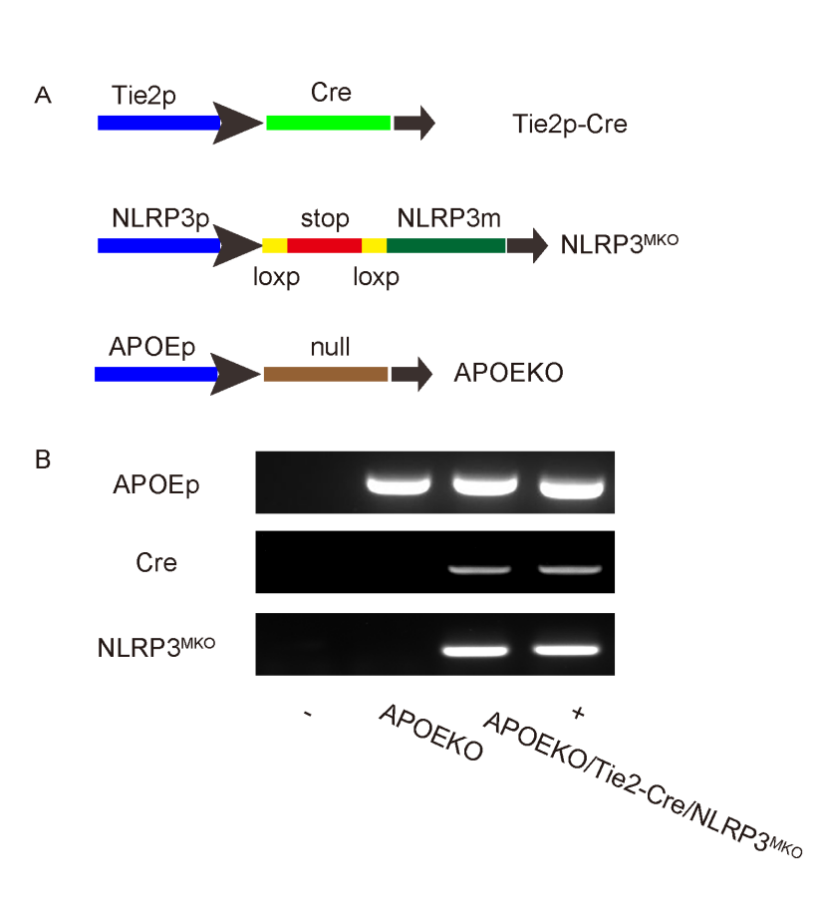
**Preparation of endothelia-specific NLRP3 mutant mice in ApoE-null background.** (**A**) Endothelia-specific NLRP3 mutant mice were prepared in ApoE-null background. Tie2 is an endothelia-specific marker. Tie2p-Cre and conditional NLRP3 mutation mice were first bred to each other to generate endothelia-specific NLRP3 mutant mice Tie2p-Cre/NLRP3^MKO^, and then bred to ApoE (-/-) mice to generate APOEKO/Tie2p-Cre/NLRP3^MKO^ mice. ApoE (-/-) mice were used as controls (APOEKO). (**B**) Representative genotyping. -, negative control. +, positive control.

In order to evaluate whether NLRP3 activation specifically in endothelial cells may cause AS in vivo, we prepared endothelia-specific NLRP3 mutant mice in ApoE-null background. Tie2 is an endothelia-specific marker. Tie2p-Cre and conditional NLRP3 mutant mice were first bred to each other to generate endothelia-specific NLRP3 mutant mice, Tie2p-Cre/NLRP3^MKO^, and then bred to ApoE (-/-) mice to generate APOEKO/Tie2p-Cre/NLRP3^MKO^ mice. ApoE (-/-) mice were used as controls (APOEKO) (Figure 3A). Genotyping was performed to confirm that genotype of these mice (Figure 3B).

### Endothelia-specific NLRP3 knockout attenuates severity of AS in HFD-treated mice

**Figure 4:**
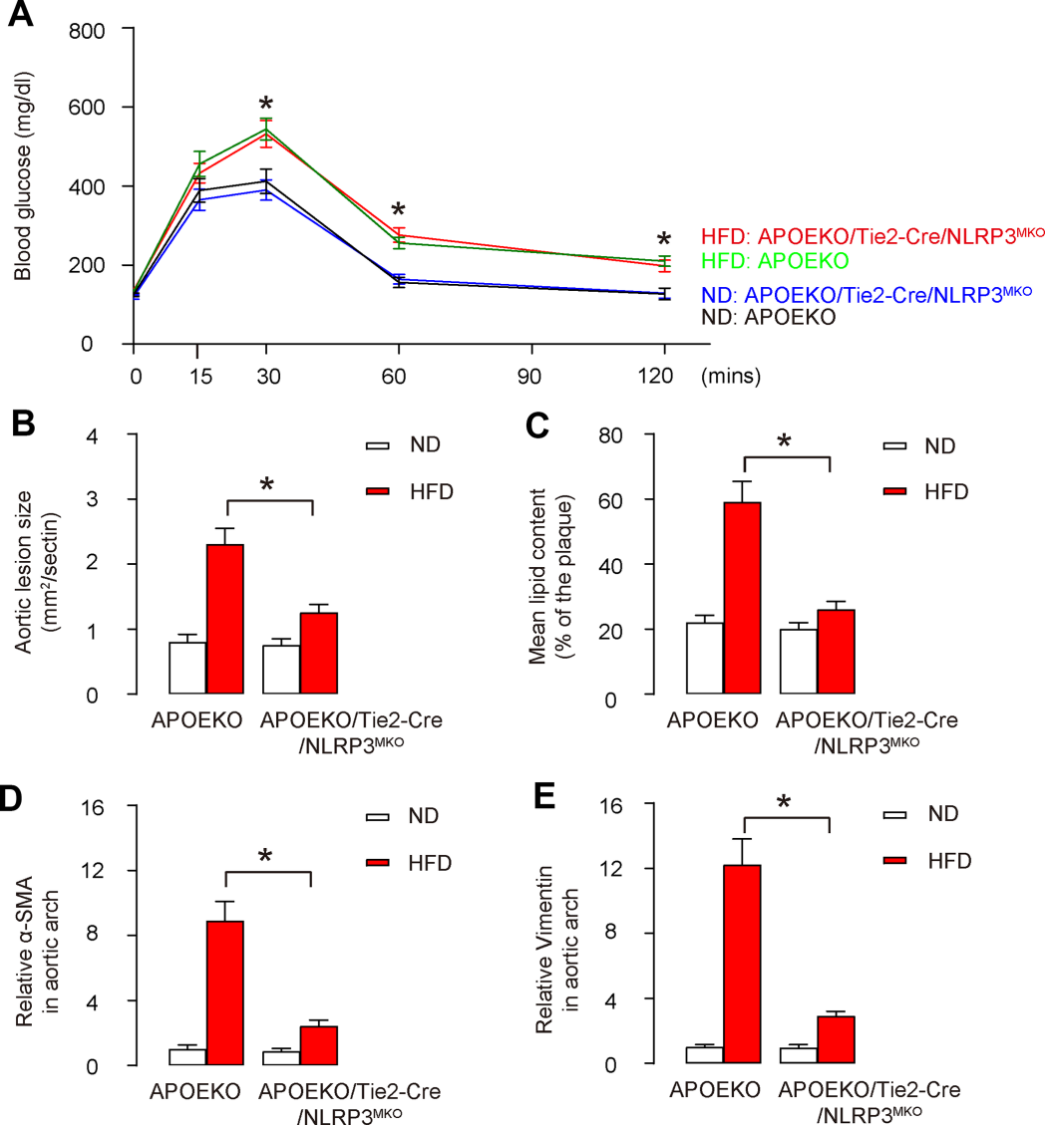
**Endothelia-specific NLRP3 knockout attenuates severity of AS in HFD-treated mice.** The animals were randomly divided into 4 groups: APOEKO with normal-diet group (ND), APOEKO with high-fat diet (HFD) group, APOEKO/Tie2p-Cre/NLRP3^MKO^ with ND, APOEKO/Tie2p-Cre/NLRP3^MKO^ with HFD group. ND or HFD treatment in mice started at 8 weeks of age, and mice were analyzed for AS lesions at 20 weeks of age. (**A**) Intraperitoneal Glucose Tolerance Testing. (**B**) Quantification of aortic lesion size by H&E-staining on aortic sinus. (**C**) Assessment of lipid content by Oil-red-O-staining on aortic sinus. (**D**, **E**) The aortic arch was isolated for analyzing the levels of α-SMA (**D**) and Vimentin (**E**) by ELISA. *p<0.05. N=10. Scale bars are 100 μm.

The animals were randomly divided into 4 groups: APOEKO with normal-diet group (ND), APOEKO with high-fat diet (HFD) group, APOEKO/Tie2p-Cre/NLRP3^MKO^ with ND, APOEKO/Tie2p-Cre/NLRP3^MKO^ with HFD group. ND or HFD treatment in mice started at 8 weeks of age, and mice were first analyzed for glucose response at 20 weeks of age, showing that HFD induced glucose intolerance in both strains, while ND did not (Figure 4A). Moreover, in control APOEKO mice, H&E-stained aortic sinus displayed a significant increase in aortic lesion size in HFD-treated mice, compared to ND-treated mice (Figure 4B). On the other hand, ND-treated APOEKO/Tie2p-Cre/NLRP3^MKO^ mice did not alter aortic lesion size compared to ND-treated APOEKO mice, while HFD-treatment-induced increases in aortic lesion size in APOEKO/Tie2p-Cre/NLRP3^MKO^ mice were significantly attenuated compared to APOEKO mice (Figure 4B). Moreover, Oil-red-O-stained aortic sinus in HFD-treated control APOEKO mice displayed a significant increase in lipid content compared to ND-treated APOEKO mice (Figure 4C). On the other hand, ND-treated APOEKO/Tie2p-Cre/NLRP3^MKO^ mice did not alter lipid content compared to ND-treated APOEKO mice, while HFD-treatment-induced increases in lipid content in APOEKO/Tie2p-Cre/NLRP3^MKO^ mice were significantly attenuated compared to APOEKO mice (Figure 4C). The aortic arch was then isolated for assessing the levels of mesenchymal markers α-SMA and Vimentin by ELISA. We detected significantly higher levels of α-SMA and Vimentin in HFD-treated APOEKO mice than ND-treated APOEKO mice, and these increases in both α-SMA and Vimentin were significantly attenuated in in HFD-treated APOEKO/Tie2p-Cre/NLRP3^MKO^ mice (Figure 4D, 4E). Together, these data suggest that endothelia-specific NLRP3 depletion attenuates severity of AS in HFD-treated mice**.**

### Endothelial cell apoptosis and ROS production are attenuated in HFD-treated endothelia-specific NLRP3 knockout mice

**Figure 5:**
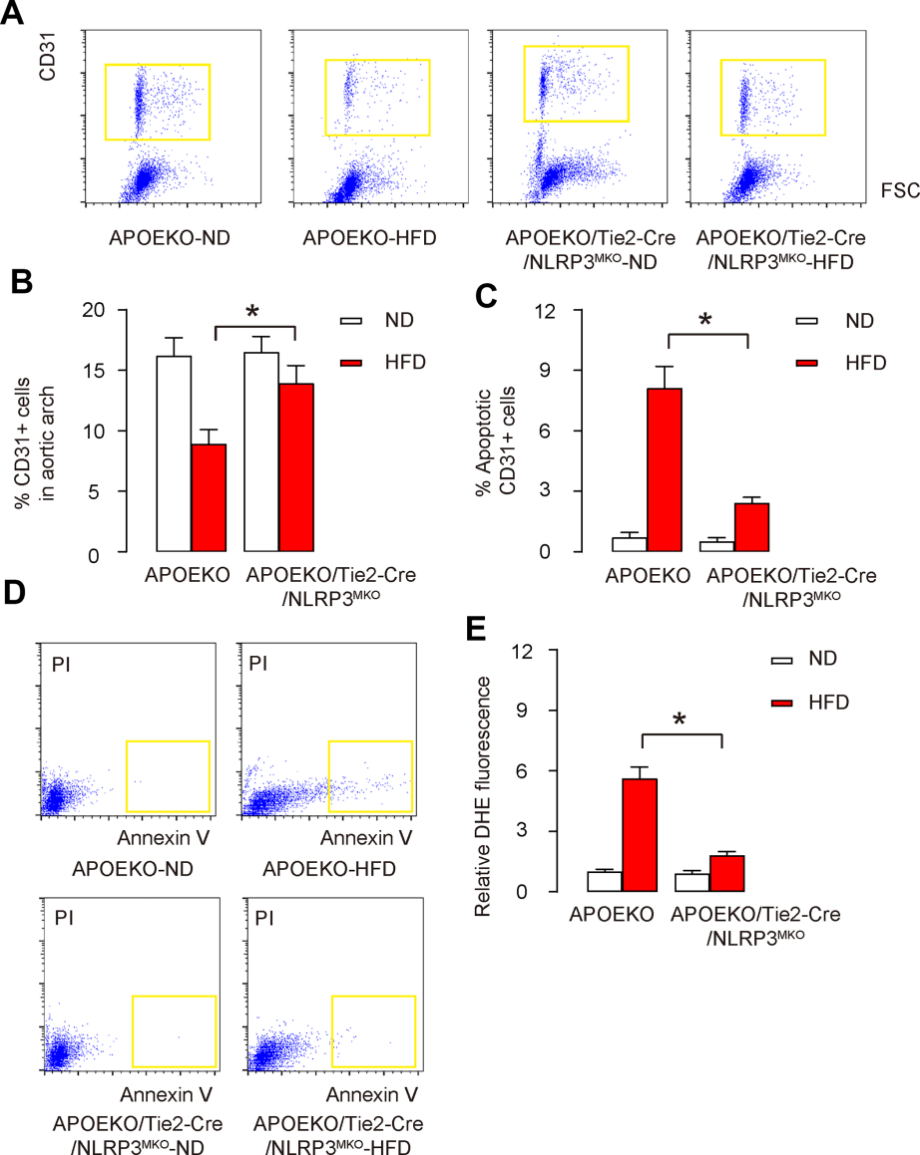
**Endothelial cell apoptosis and ROS production are attenuated in HFD-treated endothelia-specific NLRP3 knockout mice.** (**A**, **B**) CD31+ cells were isolated from the dissociated mouse aortic arch by flow cytometry, shown by representative flow charts (**A**), and by quantification (**B**). (**C**, **D**) The apoptosis of isolated CD31+ cells were analyzed by FITC Annexin V Apoptosis assay, shown by quantification (**C**), and by representative flow charts (**D**). (**E**) DHE assay for ROS. *p<0.05. N=10.

To specifically study the endothelial cells in aortic arch, we isolated CD31+ endothelial cells from the dissociated mouse aortic arch by flow cytometry (Figure 5A). We found that the percentage of CD31+ endothelial cells in the aortic arch from HFD-treated APOEKO mice was significantly lower than those from ND-treated APOEKO mice (Figure 5A, 5B). The percentage of CD31+ endothelial cells in the aortic arch from ND-treated APOEKO/Tie2p-Cre/NLRP3^MKO^ mice did not differ from ND-treated APOEKO mice, while the percentage of CD31+ endothelial cells in the aortic arch from HFD-treated APOEKO/Tie2p-Cre/NLRP3^MKO^ mice was significantly attenuated compared to HFD-treated APOEKO mice (Figure 5A, 5B). The isolated CD31+ endothelial cells were further subjected to FITC Annexin V Apoptosis essay. We detected significantly higher percentage of apoptotic CD31+ endothelial cells in HFD-treated APOEKO mice, compared to ND-treated APOEKO mice, shown by quantification (Figure 5C), and by representative flow charts (Figure 5D). The percentage of apoptotic CD31+ endothelial cells in ND-treated APOEKO/Tie2p-Cre/NLRP3^MKO^ mice did not differ from ND-treated APOEKO mice, while the percentage of apoptotic CD31+ endothelial cells in the aortic arch from HFD-treated APOEKO/Tie2p-Cre/NLRP3^MKO^ mice was significantly attenuated compared to HFD-treated APOEKO mice (Figure 5C, 5D). ROS production was also assessed. In control APOEKO mice, HFD induced significant increases in ROS production in CD31+ endothelial cells (Figure 5E). On the other hand, ND-treated APOEKO/Tie2p-Cre/NLRP3^MKO^ mice did not alter ROS production compared to ND-treated APOEKO mice, but increases in ROS production in HFD-treated APOEKO/Tie2p-Cre/NLRP3^MKO^ mice were significantly attenuated compared to HFD-treated APOEKO mice (Figure 5E). Together, these data suggest that endothelial cell apoptosis and ROS production are both attenuated in HFD-treated endothelia-specific NLRP3 knockout mice.

### NLRP3/ caspase-1/IL-1β are depleted in endothelial cells in APOEKO/Tie2p-Cre/NLRP3^MKO^ mice

**Figure 6:**
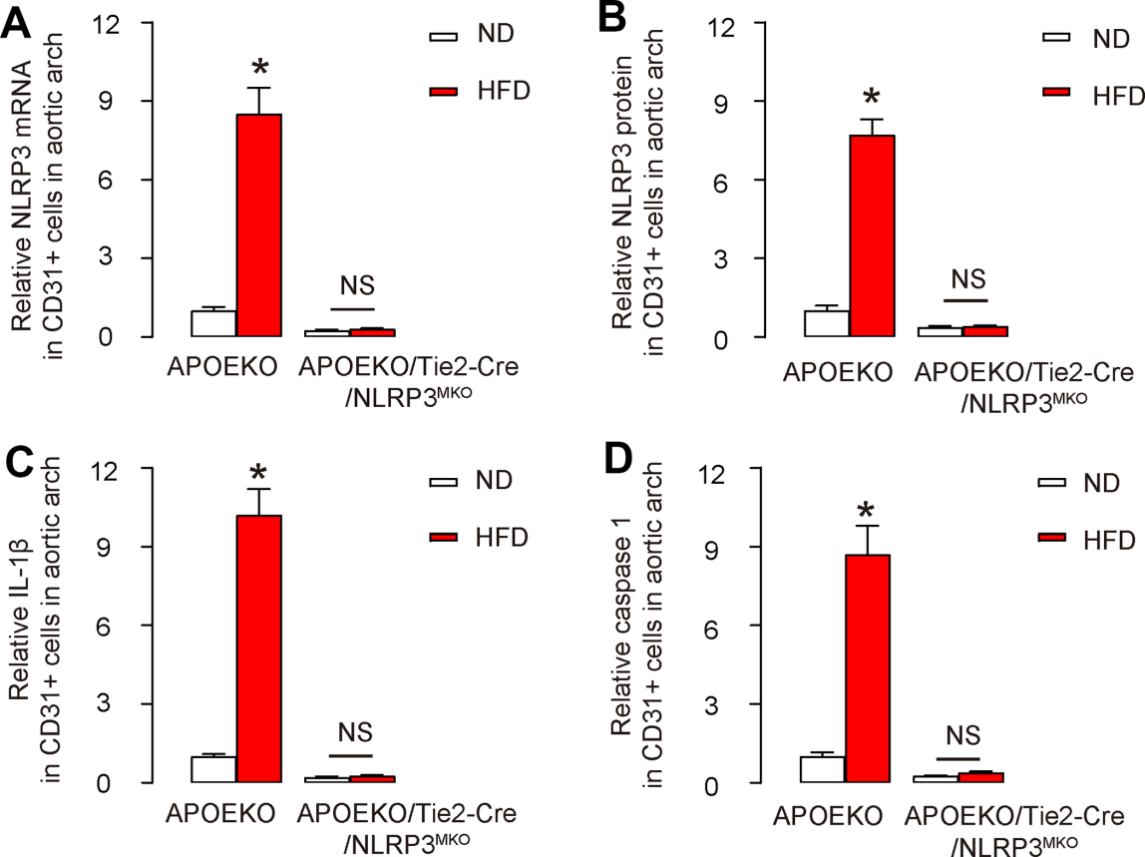
**NLRP3/ caspase-1/IL-1β are depleted in endothelial cells in APOEKO/Tie2p-Cre/NLRP3^MKO^ mice.** The knockdown of NLRP3/ caspase-1/IL-1β cascades in APOEKO/Tie2p-Cre/NLRP3^MKO^ mice was confirmed on CD31+ endothelial cells isolated from mouse aortic arch. (**A**, **B**) NLRP3 levels by RT-qPCR (**A**) and by ELISA (**B**). (**C**, **D**) IL-1β (**C**) and caspase 1 (**D**) levels by ELISA. *p<0.05. NS: non-significant. N=5.

Finally, we assessed the knockdown of NLRP3/caspase-1/IL-1β cascades in APOEKO/Tie2p-Cre/NLRP3^MKO^ mice. We found that both NLRP3 mRNA and protein levels decreased by more than 70% in aortic endothelial cells from ND-treated APOEKO/Tie2p-Cre/NLRP3^MKO^ mice compared to ND-treated APOEKO mice (Figure 6A, 6B). HFD significantly increased the NLRP3 levels in aortic endothelial cells from APOEKO mice, but these HFD-induced increases in NLRP3 levels in aortic endothelial cells were significantly attenuated in APOEKO/Tie2p-Cre/NLRP3^MKO^ mice (Figure 6A, 6B). Similar expression pattern was detected in both IL-1β (Figure 6C) and in caspase 1 (Figure 6D). These data validated the knockdown of NLRP3 in APOEKO/Tie2p-Cre/NLRP3^MKO^ mice.

## DISCUSSION

Formation of atherosclerotic lesions is mediated by a local inflammation in the vascular wall that is induced by dyslipidemia, high LDL-cholesterol and high remnant lipoprotein, all of which are boosted in a diabetic status. Endothelial dysfunction is an early characteristic for AS, since presence of endothelial cell dysfunction and injury occurs before structural alterations to the vessel wall [14–18]. IL-1β plays a key role in AS, while its active form is primarily generated by inflammasome through caspase 1 [5]. NLRP3 is the most important member for the formation and assembly of inflammasome complex [7], and has been shown to play a critical role in the development of AS. In a cohort study on the relationship among diabetes, AS and NLRP3 activation in human patients [19], the authors found that the expression of NLRP3 pathway genes was significantly higher in patients with diabetes. Moreover, activity of NLRP3 inflammasome pathway was significantly increased in patients with AS and diabetes at the early stage of plaque formation [19].

The role of NLRP3 activation in AS development has been acknowledged by some previous studies. For example, Duewell et al. showed that that minute cholesterol crystals were present in early diet-induced atherosclerotic lesions, which activated the caspase-1-activating NLRP3 inflammasome for generating active IL-1β in inflammatory cells [20]. However, the role of endothelial NLRP3 was not addressed in this study. In another study, Zheng et al. showed that silence of NLRP3 by shRNA suppressed AS progression and stabilized plaques in APOEKO mice [21]. Similarly, Wan et al. recently showed that NLRP3 activation promoted development of AS, suggesting that the function of NLRP3 in AS may be largely attributable to the endothelia-originated NLRP3 [22]. However, the strategy in both studies that injected viruses carrying a shRNA for NLRP3 driven by a ubiquitous promoter could not exclude that the experimental outcome might result from NLRP3 knockdown on non-endothelial cells, like macrophages [22]. Here, we did in vitro studies on endothelial cells, and performed in vivo studies using endothelia-specific NLRP3 knockout mice. Tie2, where 'Tie' is an acronym from tyrosine kinase with Ig and EGF homology domains, is one of the two members of the Tie family of tyrosine kinase receptors. Tie2 has highly conserved sequence across vertebrate species, with greatest amino acid homology occurring in the kinase domain, and predominantly express on the surface of endothelial cells [23]. To the best of our knowledge, our studies should be the first one to specifically study the role of NLRP3 in endothelial cells in the progression of AS.

The in vitro study here applied human endothelial cells, while for in vivo studies, a mouse model for AS was used. Although this established mouse model have many advantages in, it could not completely recapitulate all pathological changes in human AS [24]. Since each animal model has its own advantages and disadvantages, future work may use additional animal models to validate the conclusion here, in order to extrapolate the current findings into clinical studies. To summarize, our study identified a specific role for endothelial NLRP3 inflammasome in the regulation of AS development, and may shed new insight into the development of innovative AS therapy.

## MATERIALS AND METHODS

### Animal models

Tie2p-Cre (#008863) [25], conditional NLRP3 mutation (#017969) [26] and ApoE (-/-) (#002052) [27] mice were all purchased from Jackson Laboratory (Bar Harbor, ME, USA) and maintained under sterile conditions. Tie2p-Cre and conditional NLRP3 mutation mice were first bred to each other to generate endothelia-specific NLRP3 mutant mice Tie2p-Cre/NLRP3^MKO^, and then bred to ApoE (-/-) mice to generate APOEKO/Tie2p-Cre/NLRP3^MKO^ mice. ApoE (-/-) mice were used as controls (APOEKO). Female and male mice were randomly and evenly distributed in each experimental group and did not shown intra-group difference in the experimental tests. Mice at 20 weeks of age were subjected to analysis for AS development. The animals were randomly divided into 4 groups: APOEKO with normal-diet group (ND), APOEKO with high-fat diet (HFD) group, APOEKO/Tie2p-Cre/NLRP3^MKO^ with ND, APOEKO/Tie2p-Cre/NLRP3^MKO^ with HFD group.

### Quantification of atherosclerotic lesions

Mouse aortic arch was dissected out and then fixed in 4% paraformaldehyde for 4 hours, paraffin-embedded, followed by sectioning at 5μm thickness. AS lesions were examined by H&E staining and Oil red O staining (Oil red O staining kit, Abcam, Cambridge, MA, USA).

### Cell culture and transfection

Human aortic endothelial cells (HAECs, American Type Culture Collection, Rockville, MD, USA) were cultured in Endothelial Cell Medium suppled with endothelial cell growth factors, 5% fetal bovine serum (FBS, Invitrogen, CA, Carlsbad, USA) and 1% penicillin/streptomycin (Invitrogen) at 37°C with 5% CO_2_. HAECs were transfected with either a scramble sequence (scr), or a short hairpin small interfering RNA for NLRP3 (5’-CCATACCTTCAGTCTTGTCTT -3’; RiboBio Co., Ltd., Shanghai, China), using Lipofectamine 3000 reagent (Invitrogen). All plasmids also carried a red fluorescent protein (RFP). The transfected cells were purified by flow cytometry based on RFP.

### Ox-LDL treatment of the cells

The HAECs were treated with or without 100μg/ml oxidized low-density lipoprotein (ox-LDL, Beijing Xiesheng Bio-Technology Limited, Beijing, China), after which the cells underwent flow cytometry or protein/RNA extraction.

### Cell viability by cell counting kit-8 (CCK-8) assay

Cell viability was measured with an CCK-8 detection kit (Sigma-Aldrich, St. Louis, MO, USA), according to the manufacturer’s instructions.

### Apoptosis assay and flow cytometry

The cultured cells or dissociated aortic cells were re-suspended at a density of 10^6^ cells/ml in PBS. After double staining with FITC-Annexin V and propidium iodide (PI) from a FITC Annexin V Apoptosis Detection Kit I (Becton-Dickinson Biosciences, San Jose, CA, USA), cells were analyzed using a FACScan flow cytometer (Becton-Dickinson Biosciences) for determination of apoptotic cells based on expression of Annexin V and Propidium Iodide (PI). For analyses and isolation of CD31+ cells, the dissociated aortic cells were incubated with APC-cy7-CD31 (Becton-Dickinson Biosciences).

### Edu staining

Cell proliferation was determined using an Edu staining assay. Briefly, 50μmol/l EdU solution was prepared by diluting EdU solution (Solarbio Life Sciences, Beijing, China) with cell culture medium (1:1000). The treated endothelial cells were placed into 24-well plates and incubated with 100μl 50μmol/l EdU solution for 0.5 hour. After washing, the cells were fixed with methyl alcohol for 15 minutes and then decolored with 50μl 2mg/mL glycine for 5 minutes, after which the stained cells were examined.

### RT-qPCR

Total RNA was isolated with a RNeasy mini kit (Qiagen, Hilden, Germany). The extracted RNA was reverse transcribed into cDNA using High-Capacity cDNA Reverse Transcription Kit (Applied Biosystems, Foster City, CA, USA). The first-strand cDNA was used for real-time PCR to quantify mRNA expressions using QuantiTect SYBR Green PCR Kit (Qiagen). All primers were purchased from Qiagen. Data were collected and analyzed using 2-ΔΔCt method for quantification of the relative mRNA expression levels. NLRP3 values in experimental groups were sequentially normalized against GAPDH and experimental controls.

### ELISA

The cells were lysed with RIPA buffer containing protease and phosphatase inhibitors (cOmplete ULTRA Tablets, Roche, Nutley, NJ, USA). After centrifugation, the supernatant was collected and quantified for protein. ELISA for NLRP3, IL-1β, caspase-1, α-SMA and Vimentin used specific kits (R&D System, Los Angeles, CA, USA) as instructed.

### DHE assays for ROS

ROS levels in cells or aortic tissue were measured using a dihydroethidium (DHE) assay. Briefly, cells or tissue were preincubated with 20 μmol/l DHE for 1 hour, treated with alkylating agents, after which the red fluorescence was monitored at Ex/Em 520/610nm by Fluorometer (Thermo Fisher, Waltham, MA, USA). Delta fluorescence over a 12-hour period was calculated, normalized to cellular DNA content, and then expressed as % fluorescence compared with controls.

### Statistical analyses

The data in this study are shown as the mean ± S.D. Differences among groups were analyzed using one-away ANOVA with a Bonferroni correction, followed by Fisher’ Exact Test for comparison of two groups (GraphPad Prism, GraphPad Software, Inc. La Jolla, CA, USA). p < 0.05 was considered significant.

### Ethics statement

The study was approved by the Animal Care and Use Committee of Fudan University. All experimental procedures were performed in accordance with the Guide for the Care and Use of Laboratory Animals, published by the US National Institutes of Health. All experiments were conducted under the supervision of the facility’s Institutional Animal Care and Use Committee according to an Institutional Animal Care and Use Committee–approved protocol.
